# Triplet therapy for metastatic castration‐sensitive prostate cancer: Rationale and clinical evidence

**DOI:** 10.1111/iju.15647

**Published:** 2024-12-09

**Authors:** Hiroyoshi Suzuki, Shusuke Akamatsu, Masaki Shiota, Haruka Kakiuchi, Takahiro Kimura

**Affiliations:** ^1^ Department of Urology Toho University Sakura Medical Center Chiba Japan; ^2^ Department of Urology Nagoya University Nagoya Japan; ^3^ Department of Urology Kyushu University Fukuoka Japan; ^4^ Oncology Medical Affairs, Medical Affairs and Pharmacovigilance Bayer Yakuhin Ltd. Osaka Japan; ^5^ Department of Urology The Jikei University School of Medicine Tokyo Japan

**Keywords:** androgen receptor signaling inhibitor, docetaxel, metastatic castration‐sensitive prostate cancer, prostate cancer, triplet therapy

## Abstract

Prostate cancer (PC) growth is hormone‐dependent and it frequently develops distant metastases as disease progresses. Patients with metastatic castration‐sensitive prostate cancer (mCSPC) initially respond to androgen deprivation therapy (ADT) but eventually become refractory and develop metastatic castration‐resistant prostate cancer (mCRPC). Castration‐resistance is associated with high lethality and metastases confer poor prognosis, therefore unmet needs in treatment for mCSPC remain high. So far, improvements in survival in mCSPC have been achieved by doublet combination therapy such as docetaxel or an androgen‐receptor signaling inhibitor (ARSI) in addition to ADT. Further, recent phase 3 trials have shown that triplet therapy—a combination of ARSI, docetaxel, and ADT improves prognosis compared with docetaxel plus ADT in mCSPC. PC tumors manifest intra‐ and inter‐tumoral heterogeneity at both the genetic and phenotypic level. As heterogeneity increases during sequential treatment and disease progression, it is reasonable to initiate combination therapy using drugs with different mechanisms of action early in the course of disease, such as mCSPC. Previous research about tumor heterogeneity and drug resistant mechanism support this rationale, as well as preclinical studies and real‐world data provide the scientific evidence of benefit by combining ARSI and docetaxel. Here, we review the rationale and clinical evidence for triplet therapy in patients with mCSPC.

**Abbreviations & Acronyms**
ADT: androgen deprivation therapy
AR: androgen receptor
ARPC: androgen receptor pathway prostate cancer
ARSI: androgen receptor signaling inhibitor
mCRPC: metastatic castration‐resistant prostate cancer
mCSPC: metastatic castration‐sensitive prostate cancer
MOAs: modes of action
MSPC: mesenchymal and stem‐like prostate cancer
NEPC: neuroendocrine prostate cancer
OS: overall survival
PDX: patient‐derived xenograft

## INTRODUCTION

Globally, prostate cancer is the second most common cancer diagnosed in men and one of the leading causes of cancer‐associated death in men, with estimated annual prostate cancer deaths exceeding 350 000 worldwide.^1^, ^2^

Growth of benign and malignant prostate tumors is mediated by the androgen receptor (AR). Metastatic hormone‐sensitive prostate cancer (mCSPC) refers to metastatic prostate cancer that is responsive to, or has not been treated with, anti‐androgen therapy. mCSPC develops mainly by two routes: de novo (also known as synchronous disease i.e., having metastases at the time of mCSPC detection) and recurrent (or metachronous disease i.e., received prior local therapy with curative intent).^3^, ^4^ De novo mCSPC accounts for 5%–15% of all initial prostate cancer diagnoses and has poorer outcomes compared with recurrent mCSPC.^3^, ^4^, ^5^

Androgen deprivation therapy (ADT) is a standard therapy in the management of patients with prostate cancer, including mCSPC. Although patients with mCSPC initially respond to ADT, they become refractory and develop metastatic castration‐resistant prostate cancer (mCRPC), which has a poor prognosis with a median overall survival (OS) from mCRPC diagnosis of about 2–3 years.^6^, ^7^, ^8^, ^9^, ^10^ Resistance to ADT develops despite low circulating levels of testicular androgens and normal levels of adrenal androgens.

To date, combination therapy with ADT plus docetaxel or an androgen‐receptor signaling inhibitor (ARSI) has been approved worldwide and has led to improvements in survival. The prognosis for men with mCSPC has been improved by using ADT concomitantly with either docetaxel,^11^, ^12^, ^13^, ^14^ or second‐generation ARSIs: abiraterone,^15^, ^16^, ^17^, ^18^ apalutamide,^19^, ^20^ or enzalutamide.^21^, ^22^, ^23^ Randomized controlled trials (RCTs) for mCSPC showing the benefit of doublet systemic combination therapy compared to ADT alone are summarized in Table 1. Docetaxel, abiraterone, apalutamide, and enzalutamide are approved for the treatment of mCSPC in many countries including the United States (US), European Union (EU), and Japan.^24^, ^25^ However, despite major improvements in the systemic treatment of advanced prostate cancer, there remains some mCSPC patients with poor prognosis, suggesting the level of unmet needs associated with mCSPC remains high.

**TABLE 1:** Randomized controlled trials for metastatic castration‐sensitive prostate cancer with doublet systemic combination therapy.

Trial [Reference]	Patients enrolled (*n*)	Arm		Prior/concurrent docetaxel:	Median follow‐up (months)	Median OS (months)		HR (95% CI)	*p*
		Intervention	Control			Intervention	Control		
CHAARTED [13]	790	ADT + docetaxel	ADT	None	53.7	57.6	47.2	0.72 (0.59–0.89)	0.0018
STAMPEDE (M1 subgroup) [14]	1086	ADT + docetaxel	ADT	None	78.2	59.1	43.1	0.81 (0.69–0.95)	0.003
LATITUDE [17]	1199	ADT + abiraterone + prednisone	ADT + placebo	None	51.8	53.3	36.5	0.66 (0.56–0.78)	<0.0001
STAMPEDE [18]	1003	ADT + abiraterone + prednisone	ADT	None	73	79	46	Overall: 0.60 (0.50–0.71)	Overall: <0.001
								Low‐risk: 0.55 (0.41–0.76) High‐risk: 0.54 (0.43–0.69)	
ENZAMET [30]	1125	ADT + enzalutamide	ADT + NSAA	Concurrent docetaxel: 43%	68	NR	NR	Overall: 0.70 (0.58–0.84)	Overall: <0.0001
								Early docetaxel: 0.82 (0.63–1.06) No early docetaxel: 0.60 (0.47–0.78)	
ARCHES [23]	1150	ADT + enzalutamide	ADT + placebo	Prior docetaxel: 18%	44.6	NR	NR	Overall: 0.66 (0.53–0.81)	Overall: <0.001
								Prior docetaxel: 0.74 (0.46–1.20) No prior docetaxel: 0.64 (0.51–0.81)	
TITAN [20]	1052	ADT + apalutamide	ADT + placebo	Prior docetaxel: 11%	44	NR	52.2	Overall: 0.65 (0.53–0.79)	Overall: <0.0001
								Prior docetaxel: 1.12 (0.59–2.12) No prior docetaxel: 0.61 (0.50–0.76)	

Abbreviations: ADT, androgen deprivation therapy; CI, confidence interval; HR, hazard ratio; NR, not reached; NSAA, non‐steroidal antiandrogen; OS, overall survival.

Recent RCTs have shown that triplet therapy—a combination of ARSI, docetaxel, and ADT is effective in mCSPC. These include the PEACE‐1 trial of ADT plus docetaxel plus abiraterone and the ARASENS trial of ADT plus docetaxel plus darolutamide (Table 2).^26^, ^27^, ^28^, ^29^, ^30^ Darolutamide in combination with ADT and docetaxel is approved in some countries including the US, the EU, Japan, and China for mCSPC.^31^, ^32^

**TABLE 2:** Randomized controlled trials for metastatic castration‐sensitive prostate cancer with triplet systemic combination therapy.

Trial [Reference]	Patients enrolled (*n*)	Arm		Synchronous (de novo) disease (%)	High‐volume disease (%)	Median follow‐up (months)	Median OS (months)		OS HR (95% CI)
		Intervention	Control				Intervention	Control	
ARASENS [28, 29]	1306	ADT + docetaxel + darolutamide	ADT + docetaxel + placebo	86	77	43.7	NR	48.9	Overall: 0.68 (0.57–0.80)
PEACE‐1 (docetaxel subgroup) [26, 27]	710 (docetaxel subgroup)	SOC (ADT with/without docetaxel) + abiraterone; with/without RT	SOC (ADT with/without docetaxel); with/without RT	100	64	45.6	NR	53.2	Docetaxel subgroup: 0.75 (0.59–0.95)
ENZAMET (docetaxel subgroup) [30]^a^	503 (docetaxel subgroup)	ADT + docetaxel + enzalutamide	ADT + docetaxel + NSAA	72	71	68 (overall cohort)	Not reported	Not reported	Docetaxel subgroup: 0.82 (0.63–1.06)

Abbreviations: ADT, androgen deprivation therapy; CI, confidence interval; HR, hazard ratio; NA, not applicable; NR, not reached; NSAA, non‐steroidal antiandrogen; OS, overall survival; RT, radiotherapy; SOC, standard of care.

^a^ Although subgroup analysis of patients with/without docetaxel was preplanned in the ENZAMET study, the main purpose of the study was not to assess the efficacy and safety of triplet therapy; docetaxel subgroup data are included for interest only.

Although recent regulatory approval of new treatment regimens for mCSPC and mCRPC has updated the treatment landscape for metastatic prostate cancer,^33^ there has been little comprehensive discussion of the scientific rationale for triplet therapy in mCSPC. Here, we review the rationale and clinical evidence for combination therapy in patients with mCSPC.

## CHARACTERISTICS OF PROSTATE CANCER

**FIGURE 1:**
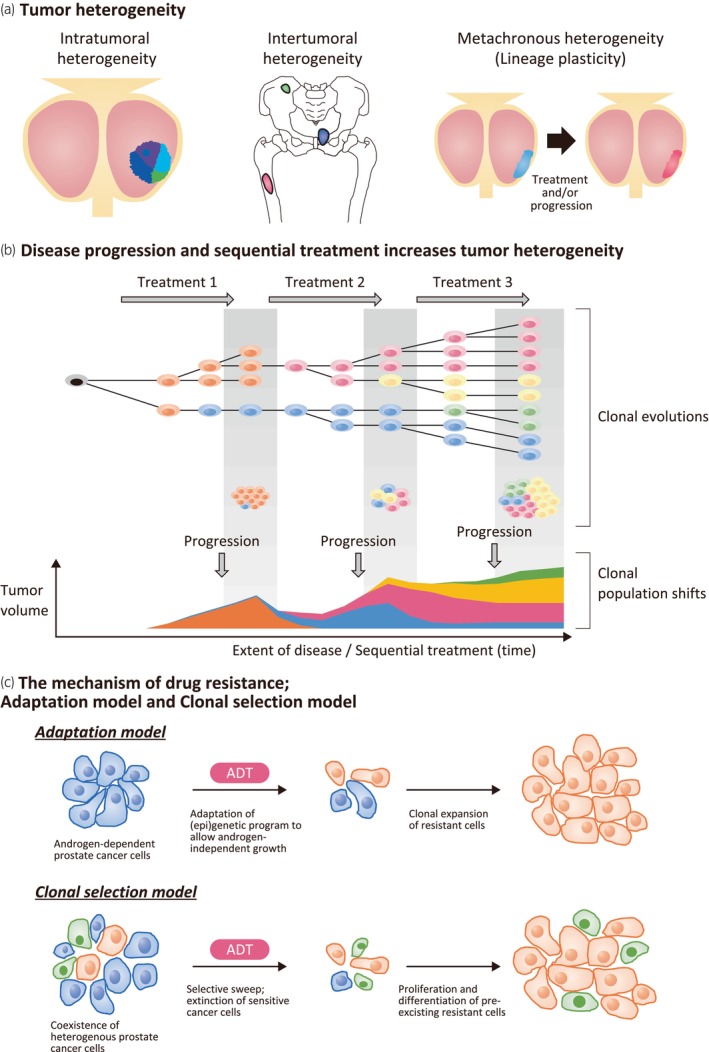
Heterogeneity of prostate cancer. (a) Tumor heterogeneity includes intratumoral heterogeneity, intertumoral heterogeneity, and metachronous heterogeneity (lineage plasticity). (b) Tumor heterogeneity increases during sequential treatment and disease progression. Clones have the potential for continued proliferation and a selective growth advantage (Darwinian selection); therefore, tumors can evolve by the expansion of one (monoclonal) or multiple (polyclonal) subpopulations to form the tumor mass. According to the later model “Darwinian selection”, genetic instability induced by disease progression or treatment pressure creates new clones (i.e., sub clone). In this manner, tumors evolve with increased heterogeneity during disease progression and sequential treatment. (c) The mechanism of drug resistance. In the Adaptation model, androgen‐dependent prostate cancer cells (blue) acquire (epi)genetic program to allow androgen‐independent growth under suppression of androgen, and the resistant cells (orange) proliferate. In the Clonal selection model, heterogenous prostate cancer cells, including androgen‐independent cells (green), coexist from an early stage of disease or at the treatment‐naïve stage. ADT/treatment induces selective sweep, causing extinction of sensitive cancer cells, and outgrowth of pre‐existing resistant cells. ADT, androgen deprivation therapy.

Strategic planning is crucial for treating prostate cancer and understanding the characteristics of prostate cancer provides the basis for treatment planning. The following sections discuss some key features of prostate cancer which lay the groundwork for considering the rationale of combination therapy. Figure 1 summarizes heterogeneity in prostate cancer, as discussed below.

### Tumor heterogeneity

Comprehensive analyzes in advanced genomic and proteomic studies in recent years have revealed the existence of tumor heterogeneity in multiple types of cancer that is more extensive than previously assumed. Tumor heterogeneity manifests at the genetic and transcriptional level, but also develop phenotypic and functional differences among cancer cells within the same tumor or intra‐patient, due to genetic and epigenetic changes, variations in the tumor microenvironment.^34^, ^35^, ^36^, ^37^ Tumor heterogeneity may promote tumor adaptation and therapeutic failure through selective pressure.^34^

Different types of tumor heterogeneity are reported, including intratumoral heterogeneity, intertumoral heterogeneity, and metachronous heterogeneity (lineage plasticity).^34^, ^38^, ^39^, ^40^, ^41^, ^42^ Intratumoral heterogeneity is defined as variations in the genomic makeup of malignant cells across distinct regions of the same tumor, and those variations between tumors with distinct anatomical locations is regarded as intertumoral heterogeneity.^43^ Generally, metachronous heterogeneity (lineage plasticity) indicates treatment‐induced changes over time in the tumor itself,^40^, ^41^, ^44^, ^45^, ^46^, ^47^ while intratumoral and intertumoral heterogeneity are known to be present in the initial stages of the disease or in treatment‐naive patients, including in localized cancer and de novo mCSPC.

In prostate cancer, there are many reports to show that primary prostate cancers demonstrate complex genomic changes with a high degree of inter‐patient, and intra‐ and inter‐tumoral heterogeneity.^38^, ^48^, ^49^, ^50^, ^51^, ^52^, ^53^, ^54^, ^55^, ^56^, ^57^, ^58^

Multifocal, localized prostate cancer is common.^59^ Genomic studies using samples from multiple spatially distinct regions from the same patient showed that multifocal, localized prostate cancer exhibits a high degree of spatial heterogeneity with different foci within the same patient displaying heterogeneity in single‐nucleotide mutations, copy number aberrations, and genomic rearrangements.^38^, ^51^, ^53^, ^54^

Also, spatial genomic studies that have sequenced multiple regions of a single tumor have suggested that most solid tumors, including primary prostate cancers, are comprised of multiple clones (termed polyclonal).^34^, ^60^, ^61^ Reconstruction of the phylogenies of 293 localized prostate cancers identified polyclonal tumors in 59% of patients. Both monoclonal and polyclonal tumors appeared to have evolved from a single ancestral cell, but a common ancestral clone diverged into subclones by acquiring mutations specific to one evolutionary branch of the tumor (i.e., subclonal mutations) in the course of cancer evolution. Specific genes (including *MTOR*, *NKX3‐1*, and *RB1*) were identified which were selectively mutated prior to, or following, subclonal diversification.^62^ Polyclonal tumors are related to prognosis and patients with polyclonal tumors have a high rate of relapse after primary therapy (61%), contrasting with patients with monoclonal tumors who have a much lower relapse rate (7%).^62^

Studies of metastatic prostate cancer also demonstrate a high level of genomic and phenotypic heterogeneity.^41^, ^42^, ^46^, ^49^, ^52^, ^55^, ^56^, ^57^, ^63^, ^64^, ^65^, ^66^, ^67^, ^68^, ^69^, ^70^, ^71^

Comprehensive genomic profiling of multiple tumor regions in individual patients revealed extensive heterogeneity within primary tumors and between primary and metastases in de novo (treatment‐naïve) mCSPC. In the study of mCSPC patients in which patients with low volume (by the criteria in the CHAARTED trial^11^) or low risk (by the criteria in the LATITUDE trial^15^), were included in over 70% of cohort, more than 60% of cases had biallelic inactivating alterations in at least one of tumor suppressor genes (*TP53, PTEN*, *or RB1*) and the prognosis of patients with these aggressive genomic profiles was poor. Furthermore, reviewing intersample concordance for alterations in these genes at primary‐site, heterogeneity for at least one gene was observed in more than 50% of cohort.^42^ These findings suggest that first, the prognosis for patients with an aggressive genomic profile is poor, even if they show a low‐risk clinical phenotype, and second, concerns about precise genotyping using a single region.

### Sequential treatment and disease progression increases tumor heterogeneity

Tumor heterogeneity plays a key role in cancer progression to metastasis. As indicated above, prostate cancer primary tumors are already heterogeneous early stage of disease or at treatment naive. Resistance to anticancer therapies can develop through selective outgrowth of resistant subclones, phenotype switching, or the emergence of a new subclone due to therapy‐induced selection.^72^

An important consideration in treatment planning is increased prostate cancer heterogeneity during sequential treatment and disease progression. Studies on the evolutionary history of prostate cancer have revealed complex patterns in metastatic disease, but much remains to be understood and requires further investigation.^41^, ^42^, ^65^, ^73^, ^74^ Gundem et al. characterized multiple metastases arising from prostate tumors in 10 patients using whole genome sequencing.^65^ Inter‐metastasis (i.e., metastasis‐to‐metastasis) spread was common, occurring either through de novo monoclonal seeding of daughter metastases or, in 50% of cases, through the transfer of multiple tumor clones between metastatic sites (i.e., polyclonal seeding). Clonal diversification occurs within the necessity to bypass ADT, driving distinct subclones toward therapeutic resistance. The study findings suggest that the more disease and metastasis progress, the more tumor complexity, including multiple clones that have acquired treatment resistance, increases.^65^ This clonal diversification in metastases has also been shown in a recent in‐depth genomic analysis of samples from patients with de novo mCSPC. The analysis showed widespread primary intra‐tumor polyclonality and discordant genotypes between primary tumors and synchronous metastases, indicating that only some primary clones which acquired the ability to metastasize actually metastasized. Additionally, the study results suggested that additional complexity was driven by polyclonal metastatic seeding from phylogenetically related primary populations.^42^

cfDNA reflects the genomic profile present in localized tissues; in addition, it contains more genomic information than tissue. Moreover, changes over time in individual patients can be tracked more easily with cfDNA than tissue. A study which tracked cfDNA from patients with mCRPC over time showed that dominant clone changed with sequential treatment/disease progression, resulting in adaptive changes against treatment and shifts of major tumor clones.^41^

Increased chromosomal and genomic instability may underlie the increase in heterogeneity caused by sequential treatment and disease progression.^71^, ^75^ A real‐world study of metastatic prostate cancer reported that the frequency of certain genomic mutations, such as aberrations in *AR, MYC*, tumor suppressor genes such as *TP53* and *RB1*, and PI3K/PTEN pathway genes, increased with treatment and disease progression. Also, certain genomic mutations and prior history of ARSI were identified as factors related to increased genome‐wide loss of heterozygosity (LOH), reflecting genomic instability.^70^ A large‐scale prospective cohort study of cancer patients, including prostate cancer, showed that chromosomal instability was strongly correlated with metastatic burden and that metastases generally had a higher fraction of clonal mutations.^71^ Another study showed that chromosomal instability results in pervasive somatic copy number alterations, which often occur in parallel throughout tumor evolution.^76^

These findings mean that sequential treatment and disease progression, including metastasis, are related to increased chromosomal and genomic instability, bringing about changes in the expression of many genes and abnormal protein function that, in turn, drive subclone diversity and further tumor evolution.

### Treatment‐resistant tumor cells are present in the earliest disease stages of prostate cancer

To date, there are some reports that treatment‐resistant cancer cells are present in the earliest stages of disease in prostate cancer.

In a small study, Cheng et al. isolated epithelial cells from prostate cancer patients, including treatment‐naive adenocarcinoma, locally recurrent CRPC, and metastatic CRPC, and classified cells into subpopulations which have different characteristics using single‐cell RNA sequencing.^77^ They revealed that CRPC‐like cells were present in treatment‐naïve patients. This finding suggests that CRPC‐like cells pre‐exist early in the development of prostate cancer, indicating that treatment‐resistance is not acquired exclusively by hormone therapy alone.^77^

Han and colleagues showed that mesenchymal and stem‐like prostate cancer (MSPC) without AR expression is present in treatment‐naïve prostate cancer.^44^ Bioinformatic analysis of patient‐derived xenografts (PDXs), cell lines, and organoids identified three intrinsic transcriptional subtypes of mCRPC, including AR‐pathway prostate cancer (ARPC), MSPC, and neuroendocrine prostate cancer (NEPC) cells. MSPC has no AR expression and features robust expression of the mesenchymal marker vimentin and/or the prostate stem cell marker ITGB4. There was partial overlap with the AR+ (ARPC), AR−/NE− (double‐negative prostate cancer; DNPC), and NE+ (NEPC) subtypes previously defined using immunohistochemistry‐based gene sets.^78^ Analysis of clinical datasets, including treatment‐naive localized prostate cancer patients, showed a certain proportion of patients not only had ARPC but also MSPC. Furthermore, the datasets of patients with mCRPC indicated that the proportion of ARPC tends to decrease with ARSI treatment; conversely, the proportion of MSPC tends to increase, indicating that MSPC arises from ARPC due to therapy‐induced lineage plasticity. Therefore, the authors suggested that MSPC can occur de novo in the primary site, or in response to hormone therapy.^44^

## RATIONALE FOR COMBINATION THERAPY

**FIGURE 2:**
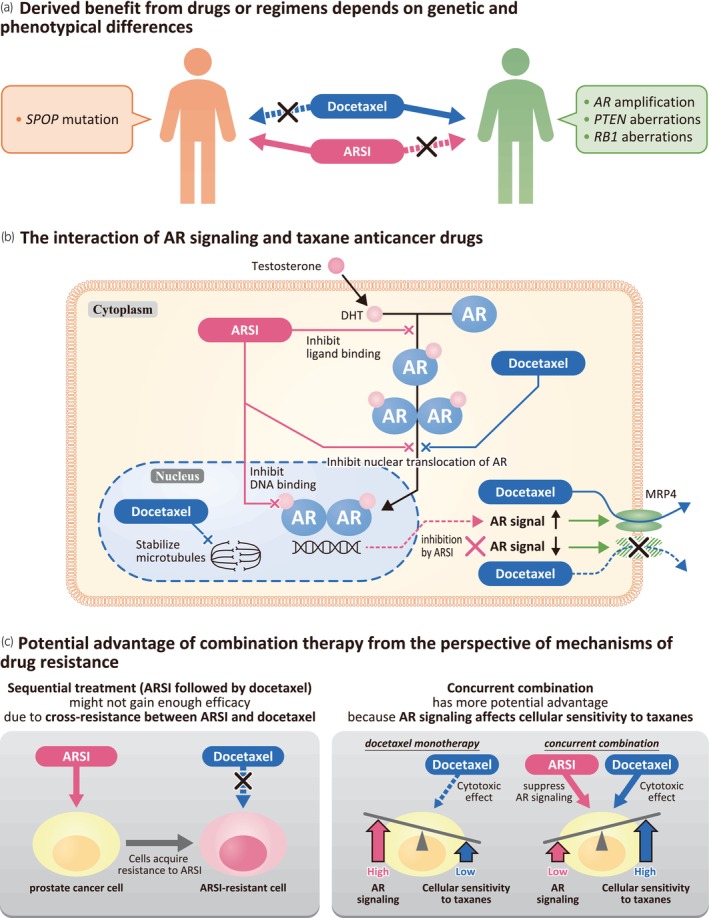
Pre‐clinical evidence for the rationale of combining ARSI and taxane anticancer drugs. (a) ARSI and docetaxel have different advantages. Real‐world studies suggest that the efficacy of taxanes is greater than that of ARSI in patients with *AR* amplification, *PTEN* aberrations or *RB1* aberrations, while the efficacy of ARSI is better than taxanes in patients with *SPOP* mutations. (b) While the primary mode of action of docetaxel is stabilization of microtubules, which results in the inhibition of mitosis in cells, docetaxel also has an action against the AR, in which it inhibits nuclear translocation of AR. Also, MRP4 is known to be involved in one of the mechanisms of docetaxel resistance. MRP4 expression is regulated by AR signaling, being upregulated by androgen. Because MRP4 expression is downregulated by ARSI, it can lead to the reduction of extracellular efflux of docetaxel, which results in restoring the cytotoxicity effect of docetaxel. (c) ARSI‐resistant cells show collateral resistance to taxanes; therefore, if docetaxel is administered sequentially after ARSI, efficacy might not be optimal. In addition, activation of AR signaling affects taxane‐sensitivity in prostate cancer cells; therefore, ARSI administration concurrently combined with docetaxel can augment the cytotoxic effect of docetaxel. These findings support the idea that the upfront use of taxanes in combination with ARSI is reasonable, rather than sequential treatment. AR, androgen receptor; ARSI, androgen‐receptor signaling inhibitor; DHT, dihydrotestosterone; MRP4, multidrug resistance protein 4.

Due to the heterogeneity in prostate cancer described above, it is reasonable to suggest that combination therapy using agents with different modes of action (MOAs) may be more effective than monotherapy or combination therapy using agents with similar MOAs. Both preclinical and real‐world studies have supported the notion that combining AR signaling inhibition with taxane anticancer drugs may provide a therapeutic benefit or enhanced effectiveness. Preclinical evidence supporting the rationale for combining ARSI and docetaxel is outlined in Figure 2.

### Derived benefit from drugs or regimens depends on genetic and phenotypical differences

First, drugs with different MOAs may have different advantages and, therefore, complement each other. A retrospective real‐world study showed differences in efficacy for ARSIs and taxane anticancer drugs in patients with mCRPC depending on the type of genetic abnormalities. Adjusted multivariable models for OS and time to next treatment, showed that patients with *AR* amplification, *PTEN* aberrations or *RB1* aberrations had a poor prognosis overall, and these three abnormalities were associated with reduced effectiveness to ARSIs but not to taxanes.^79^ In contrast, another real‐world study showed that *SPOP* mutations were associated with improved outcomes in patients with de novo mCSPC treated with combination ADT plus ARSI, but not with ADT plus docetaxel.^80^ Regarding factors other than gene mutations, Sutera and colleagues focused on differences in biological characteristics and treatment responses between patients with synchronous and metachronous mCSPC to identify patient characteristics indicative of a greater benefit from docetaxel. OS was significantly longer with combination therapy (ADT + non‐hormone therapy) compared to ADT monotherapy in synchronous patients (HR = 0.47, 95% CI 0.30–0.72, *p* < 0.01), whereas there was no difference in OS between those therapies in metachronous patients. This observation may be because patients with synchronous mCSPC had lower AR activity and androgen responsiveness than patients with metachronous disease. Therefore, patients with synchronous mCSPC may not respond sufficiently to AR‐targeting therapy alone and likely derive a greater benefit from AR‐targeting therapy plus non AR‐targeting therapy.^4^

### The interaction of AR signaling and taxane anticancer drugs

There are some reports from nonclinical studies to support the potential benefits of combining docetaxel and AR signaling blockade. The impact of testosterone and AR‐pathway stimulation on the antitumor efficacy of taxane anticancer drugs (docetaxel and cabazitaxel) was assessed in two studies using CRPC PDXs and AR‐positive CRPC cell lines.^81^, ^82^ Both docetaxel and cabazitaxel are semisynthetic taxanes which bind to and stabilize microtubules, resulting in cell‐cycle arrest and apoptosis.^83^, ^84^ Initially, the studies revealed that testosterone impairs docetaxel antitumor activity through two different mechanisms: first, by impairing docetaxel tumor accumulation, thereby reducing tubulin stabilization; and second, by activating the AR pathway, thereby interfering with docetaxel‐induced cell death. These results indicate that androgen levels and AR signaling need to be suppressed for optimizing docetaxel efficacy.^81^ Using PDX models treated with enzalutamide and cabazitaxel, Mout et al. also reported the mechanisms of the combined effect of ARSI plus taxane anticancer drugs.^82^ Adding enzalutamide to cabazitaxel induced greater anti‐tumor activity compared to enzalutamide or cabazitaxel alone, and combination treatment was found to remain active even in enzalutamide‐resistant CRPC models. This may be because AR downstream signaling was significantly impacted by enzalutamide treatment, although enzalutamide failed to impact tumor growth by itself. The study also showed that targeting the AR increased the rate of cabazitaxel‐induced apoptosis.^82^

Buck and colleagues reported the effects of combining darolutamide and docetaxel as well as the mechanisms involved. An in vivo study showed that combined treatment with darolutamide plus docetaxel increased the anti‐tumor effect compared with either drug alone in androgen‐sensitive prostate cancer organoid models, and in PDX CRPC models.^85^ The authors focused on the AR and cell‐cycle interaction when explaining the mechanism of the combined effect. Taxane anticancer drugs cause cell‐cycle arrest at the G2/M phase,^86^, ^87^ while the AR is a critical regulator of G1 to S phase transition for androgen‐dependent tumor cell proliferation.^88^ In vitro experiments showed that darolutamide induced cell‐cycle arrest at the G1‐S transition. These results suggest that blocking the AR pathway inhibits the G1‐S phase transition and further inhibits the growth of taxane‐resistant cancer cells, thereby improving the efficacy of taxane‐based chemotherapy.^85^ These preclinical results support the additional benefits derived from the combination of ARSIs and docetaxel compared with docetaxel alone observed in phase 3 studies.

- The action of docetaxel against the AR
  The action of docetaxel on the AR may also explain the effects achieved with combined ARSIs plus docetaxel. In vitro studies using prostate cancer cell lines show that taxanes inhibit ligand‐induced AR nuclear translocation and sequester AR into the cytoplasm, thereby impairing AR signaling,^89^, ^90^ and taxanes inhibit downstream signaling of AR target genes.^90^ Thus, the effects of docetaxel in combination with ARSIs may be mediated, in part, by inhibition of AR nuclear transport and signaling.
- ARSIs improve docetaxel sensitivity in cancer cells
  Additionally, using an ARSI in combination with docetaxel may contribute to improving docetaxel resistance. Multidrug resistance protein 4 (MRP4/ABCC4) is a member of the ATP‐binding cassette (ABC) transporter superfamily and is expressed in normal tissues including the prostate and kidney. The transmembrane protein MRP4 is an efflux pump that functions to transport a range of organic anionic compounds (both endogenous and xenobiotic) out of the cell and is involved in the development of multidrug resistance. MRP4 expression is associated with prostate cancer malignancy and is involved in one of the mechanisms of docetaxel resistance.^91^, ^92^, ^93^, ^94^ Li et al. reported that anti‐androgen therapy suppresses MRP4 expression and improves docetaxel sensitivity in docetaxel‐resistant cancer cells.^93^ Using two prostate cancer cell lines which displayed differential sensitivity to the taxane, Komura and colleagues showed that AR signaling significantly affects the sensitivity of prostate cancer cells to docetaxel. Genetic analysis of both cell lines revealed that differential docetaxel sensitivity was associated with KDM5D (lysine‐specific demethylase 5D) which is an epigenetic regulator of the AR axis. Reduced KDM5D expression dysregulates AR signaling, leading to a loss of docetaxel sensitivity.^95^ These results suggest that resistance to docetaxel may be delayed by the addition of an ARSI.

### Potential advantage of combination therapy from the perspective of mechanisms of drug resistance

Two main models have been proposed for drug resistance in prostate cancer: “adaptation” and “clonal selection” models (Figure 1).^96^ The adaptive model proposes that previously androgen‐dependent cells acquire hormone resistance capable of surviving in low‐androgen environments via AR pathway inhibition by treatments, suggesting an adaptive change to ADT via genetic/epigenetic conversion events. In contrast, the clonal selection model considers that pre‐existing aggressive CRPC cells with low AR activity, which have a selective advantage during ADT, grow during the treatment process.^34^, ^37^, ^97^, ^98^

Given both of these models, targeting cancer cells before they acquire resistance through adaptive change caused by treatment and attacking pre‐existing aggressive CRPC cells before they can proliferate, should be key to confronting the issue of drug resistance. Initiating combination therapy with drugs with different mechanisms of action early in prostate cancer, before castration resistance has developed, may have the potential to cover both mechanisms and increase the chances of success (Figure 1).

In addition, the close relationship between tumor heterogeneity and drug resistance may also help to explain the rationale of combination therapies. As discussed earlier, cancers become more heterogeneous during the course of disease progression.^34^, ^35^, ^36^, ^37^, ^41^, ^42^, ^65^, ^70^ Treatment has even less chance of succeeding once cancer acquires advanced heterogeneity and plasticity. Therefore, there is reasonable potential to overcome resistance by initiating combination therapy of drugs with different mechanisms from an early disease stage before tumor heterogeneity increases. Similar to this theory, Kita and colleagues have previously discussed the rationale for starting combination therapy early to address the heterogeneity of prostate cancer.^39^ Furthermore, since ARSI‐resistant cells are also resistant to docetaxel,^99^ concurrent combination therapy with ARSI and docetaxel may be more reasonable than sequential treatment with ARSI followed by docetaxel. Shiota and colleagues showed that comprehensive inhibition of AR signaling, both for normal and aberrant signaling, increases the sensitivity of cancer cells to taxanes.^99^ Therefore, it seems reasonable to use docetaxel first, at an early stage with hormone sensitivity before abnormal AR signaling presents, and to use taxanes in combination with ARSI.

## PHASE 3 CLINICAL TRIALS OF TRIPLET COMBINATION THERAPY

The PEACE‐1^26^, ^27^ and ARASENS^28^, ^29^ trials clearly demonstrate the benefit of triplet therapy over docetaxel plus ADT doublet therapy (Table 2). The PEACE‐1 trial was an open‐label RCT with a 2 × 2 factorial design, comparing abiraterone plus prednisone, with or without radiotherapy, in addition to standard of care (ADT alone or with docetaxel) in de novo mCSPC. In the docetaxel sub‐populations, triplet therapy (abiraterone, docetaxel plus ADT) significantly prolonged OS compared with docetaxel plus ADT (HR: 0.75, 95% CI 0.59–0.95).^26^ The ARASENS trial was a double‐blind RCT comparing combination darolutamide, ADT, and docetaxel with placebo, ADT, and docetaxel in patients with mCSPC (de novo and recurrent). Triplet therapy significantly prolonged OS and reduced the risk of death by 32.5% compared with docetaxel plus ADT (HR: 0.68, 95% CI 0.57–0.80).^28^

## FUTURE PROSPECTS

Given the characteristics of prostate cancer described in this review, triplet therapy for mCSPC appears to be a sensible and effective regimen, but further work is required to identify the profile of patients most likely to benefit from this regimen and the most appropriate treatments to be given after progression with triplet therapy. This can be partially achieved through real‐world experience of triplet therapy which is currently lacking. Safety management of triplet therapy in routine clinical practice also needs to be addressed. Also, because data directly comparing triplet therapy versus ARSI plus ADT are lacking, superiority of triplet therapy in comparison to ARSI plus ADT has not been established.

Due to a lack of evidence supporting the efficacy of combination therapy using drugs with different MOAs, sequential doublet therapies, a drug plus ADT, were standard in prostate cancer until recently. However, recent evidence has emerged to indicate the efficacy and safety of triplet therapy with ARSI, docetaxel, plus ADT for mCSPC and triplet therapy with PARP inhibitors, ARSI, plus ADT.^100^, ^101^, ^102^ Given the potential of combination therapies containing agents with different MOAs, the continued development of new regimens is warranted.

## CONCLUSIONS

Initiating triplet therapy with ARSI, docetaxel, and ADT early in the course of prostate cancer, such as mCSPC, is a reasonable and promising treatment option given the heterogeneous characteristics of primary prostate cancer and drug resistance mechanisms. Genomic instability and heterogeneity increase with sequential therapy, disease progression, and metastasis. More extensive preclinical and clinical research, as well as real‐world data, should further support this rationale.

## AUTHOR CONTRIBUTIONS

**Hiroyoshi Suzuki:** Writing – original draft; conceptualization; writing – review and editing. **Shusuke Akamatsu:** Conceptualization; writing – original draft; writing – review and editing. **Masaki Shiota:** Conceptualization; writing – original draft; writing – review and editing. **Haruka Kakiuchi:** Conceptualization; writing – original draft; writing – review and editing. **Takahiro Kimura:** Conceptualization; writing – original draft; writing – review and editing.

## CONFLICT OF INTEREST STATEMENT

H.S. reported receiving honoraria for lectures from Bayer Yakuhin, Ltd., Astellas Pharma Inc., AstraZeneca K.K., Janssen Pharmaceutical K.K., Ferring Pharmaceuticals Co., Ltd. and Sanofi K.K., and grants or funding from Bayer Yakuhin, Ltd., Astellas Pharma Inc., AstraZeneca K.K., Janssen Pharmaceutical K.K., MSD K.K. and Eli Lilly Japan K.K.

S.A. reported receiving honoraria from Janssen Pharmaceutical K.K., Bayer Yakuhin, Ltd., Sanofi K.K., Takeda Pharmaceutical Co., Ltd., Astellas Pharma Inc. and Pfizer Japan Inc., research grant from Tosoh Co. and advisory board for Janssen Pharmaceutical K.K., Bayer Yakuhin, Ltd., Sanofi K.K., Takeda Pharmaceutical Co., Ltd., Astellas Pharma Inc., Pfizer Japan Inc. and Novartis Pharma K.K.

M.S. reported receiving honoraria for lectures from Janssen Pharmaceutical K.K., Astellas Pharma Inc., AstraZeneca K.K. and Sanofi K.K. and grants or funding from KUBIX Inc. and Astellas Pharma Inc.

H.K. is an employee of Bayer Yakuhin Ltd.

T.K. reported receiving honoraria for lectures from Astellas Pharma Inc., AstraZeneca K.K., Sanofi K.K., Bayer Yakuhin Ltd. and Janssen Pharmaceutical K.K.

Dr. Shusuke Akamatsu is an Associate Editor of the International Journal of Urology. Dr. Hiroyoshi Suzuki and Dr. Takahiro Kimura are Editorial Board members of the International Journal of Urology. They are co‐authors of this article.

## FUNDING INFORMATION

Bayer Yakuhin Ltd.
